# Inhibition of Mnk enhances apoptotic activity of cytarabine in acute myeloid leukemia cells

**DOI:** 10.18632/oncotarget.10796

**Published:** 2016-07-23

**Authors:** Peng Li, Sarah Diab, Mingfeng Yu, Julian Adams, Saiful Islam, Sunita K.C. Basnet, Hugo Albrecht, Robert Milne, Shudong Wang

**Affiliations:** ^1^ Centre for Drug Discovery and Development, Sansom Institute for Health Research, and School of Pharmacy and Medical Sciences, University of South Australia, Adelaide, South Australia 5001, Australia

**Keywords:** Mnk inhibitor, acute myeloid leukemia, cytarabine, combination therapy

## Abstract

Cytarabine (Ara-C) is a first line clinical therapeutic agent for treatment of acute myeloid leukemia (AML). However, this therapy is limited due to high rate of resistance and relapse. Recent research has revealed that the poor prognosis and resistance to Ara-C in AML were associated with its abnormally activated MAPK pathways. In this study, we showed a strong synergistic effect of Ara-C with either our Mnk inhibitor (MNKI-8e) or short hairpin RNA (shRNA) mediated knockdown of Mnks in MV4-11 AML cells. We investigated the underlying mechanisms for this synergism. We showed that both MNKI-8e and Mnk shRNAs enhanced the ability of Ara-C to induce apoptosis. We found that Ara-C increased the phosphorylation of Erk1/2, p38 and eIF4E, which correlated with an enhanced level of anti-apoptotic Mcl-1 protein. Inhibition of Mnk activity suppressed the Ara-C-induced MAPK activity, and thus enhanced apoptosis in MV4-11 cells. Taken together, our study suggests that MAPK-Mnk-eIF4E pathway plays a critical role in Ara-C-treated MV4-11 cells and targeting Mnk may be a promising therapeutic strategy for sensitizing leukemic cells to Ara-C therapy.

## INTRODUCTION

The current treatments for acute myeloid leukemia (AML) have major limitations, and the need for novel therapies is urgent and of high clinical importance [1, 2]. The typical treatment strategy for AML, referred to as 7+3 therapy, involves a regimen of cytarabine (Ara-C) followed by daunorubicin or idarubicin [3]. These chemotherapeutic treatments have high relapse rates (about 70%) and are associated with resistance and toxicity, leading to dismal long term survival rates and poor quality of life for the patients with AML [4]. Unfortunately, the treatments for AML have not significantly improved over the past two decades.

It has recently been recognized that the activated mitogen-activated protein kinase (MAPK) pathways play important roles in the poor prognosis of AML and its development of resistance to Ara-C treatment [5–8]. The MAPK pathways control mRNA export and translation, regulate proliferation of cancer cells and drive survival and resistance to chemotherapy. Erk1/2 and p38 are key kinases of the MAPK pathways and activate MAPK-interacting kinases (Mnks), which subsequently phosphorylate the eukaryotic initiation factor 4E (eIF4E) and promote the synthesis of tumorigenic proteins [9, 10]. eIF4E is a key component of the messenger RNA (mRNA) cap-binding complex. It has been suggested that the phosphorylation of this protein by Mnks may enhance the translation of some mRNAs to produce malignancy-associated proteins, including anti-apoptotic proteins *e.g*. Mcl-1 and Bcl-2, oncoproteins *e.g*. c-Myc and cyclin D1 and inducers of angiogenesis such as VEGF [9, 11]. Elevated phosphorylation of eIF4E has been observed in many malignancies including AML [7, 12]. A recent study reported that Ara-C treatment activated the MAPK pathways by inducing phosphorylation of Erks and enhancing expression of the anti-apoptotic proteins Mcl-1 and Bcl-2 [8]. Induction of the anti-apoptotic proteins was believed to play an important role for the relapse and resistance to Ara-C therapy. As inhibition of Mnks can block the synthesis of these pro-survival proteins and sensitize apoptosis in cancer cells, along with the non-essential role of Mnks in normal development [13], Mnk inhibitors could provide an effective but less toxic therapeutic strategy to brake the Ara-C resistance in AML therapy.

Cercosporamide, an anti-fungal agent, is a non-selective Mnk inhibitor and can effectively reduce eIF4E phosphorylation [14]. Cercosporamide has been shown to enhance the anti-AML activity of Ara-C *in vitro* and *in vivo* [7]. The eIF4E inhibitor ribavirin has been shown to block the eIF4E-dependent export and translation of mRNA and to suppress tumor growth in a mouse xenograft model [12, 15]. Ribavirin also enhanced the effects of Ara-C during a clinical trial for AML treatment [16]. Despite the recognition of the important role of MAPK pathways in the resistance of AML cells to Ara-C treatment, little progress has been made to understand the underlying mechanisms.

Our group has discovered a number of highly potent and selective Mnk inhibitors which have demonstrated anti-cancer activity against a variety of cancer cell lines, including AML cells [17–20]. MNKI-8e, a derivative of 5-(2-(phenylamino)pyrimidin-4-yl)thiazol-2(3*H*)-one, inhibited Mnk2 activity, blocked phosphorylation of eIF4E, downregulated anti-apoptotic protein Mcl-1 and induced apoptosis in AML cells [17]. In the current work, MNKI-8e and short hairpin RNA (shRNA) mediated knockdown of Mnk1 and Mnk2, *i.e*. Mnk1 KD and Mnk2 KD, as well as double knockdown of Mnk1&2, *i.e*. Mnk1&2 KD, were used to investigate the mechanisms of action in AML cells. We found that MNKI-8e, as well as Mnk1&2 KD, substantially potentiated the Ara-C induced apoptosis in MV4-11 cells through inhibiting Mnk kinase activity.

## RESULTS

### MNKI-8e is a potent anti-proliferative agent and enhances the cytotoxicity of Ara-C

The anti-cancer activity of MNKI-8e was firstly assessed against five human AML cell lines, *i.e*. MV4-11, Kasumi-1, PL-21, KG-1 and U937, which represent different French–American–British (FAB) subtypes. The effects of MNKI-8e on cell viability were initially assessed with a 72 h proliferation assay as summarized in Table 1. MNKI-8e was potent against all cell lines with GI_50_ in a range of 1.23 to 7.36 μM, being most potent against MV4-11 (GI_50_ = 1.32 μM) and KG-1 (GI_50_ = 1.23 μM) cells. In contrast, MNKI-8e showed no effects against non-transformed WI-38 lung fibroblasts (GI_50_ >100 μM), suggesting its high selectivity for leukemia cells.

**Table 1:** Effects of MNKI-8e on cell viability

Cell lines	FAB subtype	GI_50_ (μM)^*^
MV4-11	M5	1.32 ± 0.40
Kasumi-1	M2	7.36 ± 1.16
PL-21	M3	7.12 ± 2.15
KG-1	M0/M1	1.23 ± 0.22
U937	M5	3.14 ± 0.30
WI-38^**^		>100^**^

* By 72 h resazurin assay, and

** 72 h MTT assay. Data given are the mean ± SD from at least two replicates

**Figure 1:**
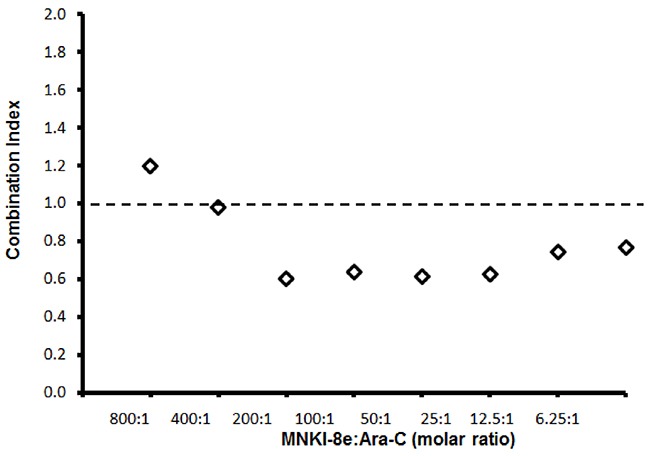
Synergistic effects of MNKI-8e and Ara-C in MV4-11 cells Combination effects of MNKI-8e and Ara-C at different molar ratios by a 72 h cytotoxicity assay and the combination indices (*i.e*. CI = 1 additive effect, CI < 1 synergy or CI > 1 antagonism) are shown. The CI values were calculated by the median-effect analysis method [21, 22] and computed using the CompuSyn program (ComboSyn, Inc.).

We next evaluated cytotoxic effects of a combined treatment of MNKI-8e with Ara-C on MV4-11 cells. MNKI-8e, at a concentration of 1 μM or 5 μM, enhanced the anti-leukemic activity of Ara-C, resulting in an over 10-fold increase in potency when compared to Ara-C treatment alone (Table 2). We used the median-effect analysis method to calculate combination indices (CIs) [21, 22] and CI values < 1.0, = 1.0 and > 1.0 indicated synergistic, additive and antagonistic effects, respectively. The CI values were calculated with the CompuSyn program (ComboSyn Inc.) using data from cell viability experiments with various concentrations of MNKI-8e versus Ara-C. As shown in Figure 1, synergistic interactions were achieved at molar ratios of MNKI-8e to Ara-C ≤ 200:1.

**Table 2:** MNKI-8e, Mnk1 KD, Mnk2 KD and Mnk1&2 KD enhanced the effects on Ara-C mediated cell viability in MV4-11 cells

Treatment to MV4-11 cells	GI_50_ of Ara-C (μM)^*^
DMSO control	0.090 ± 0.002
0.2 μM MNKI-8e	0.071 ± 0.010
1 μM MNKI-8e	0.007 ± 0.001
5 μM MNKI-8e	< 0.001
WT control^**^	0.089 ± 0.016
Mnk1 KD	0.074 ± 0.017
Mnk2 KD	0.054 ± 0.014
Mnk1&2 KD	0.003 ± 0.001

* 72 h resazurin assay in MV4-11 cell line;

** MV4-11 cell treated with empty lentiviral vectors. Data given are the mean ± SD derived from at least two replicates.

### MNKI-8e induces apoptosis without activation of caspases 3/7

**Figure 2:**
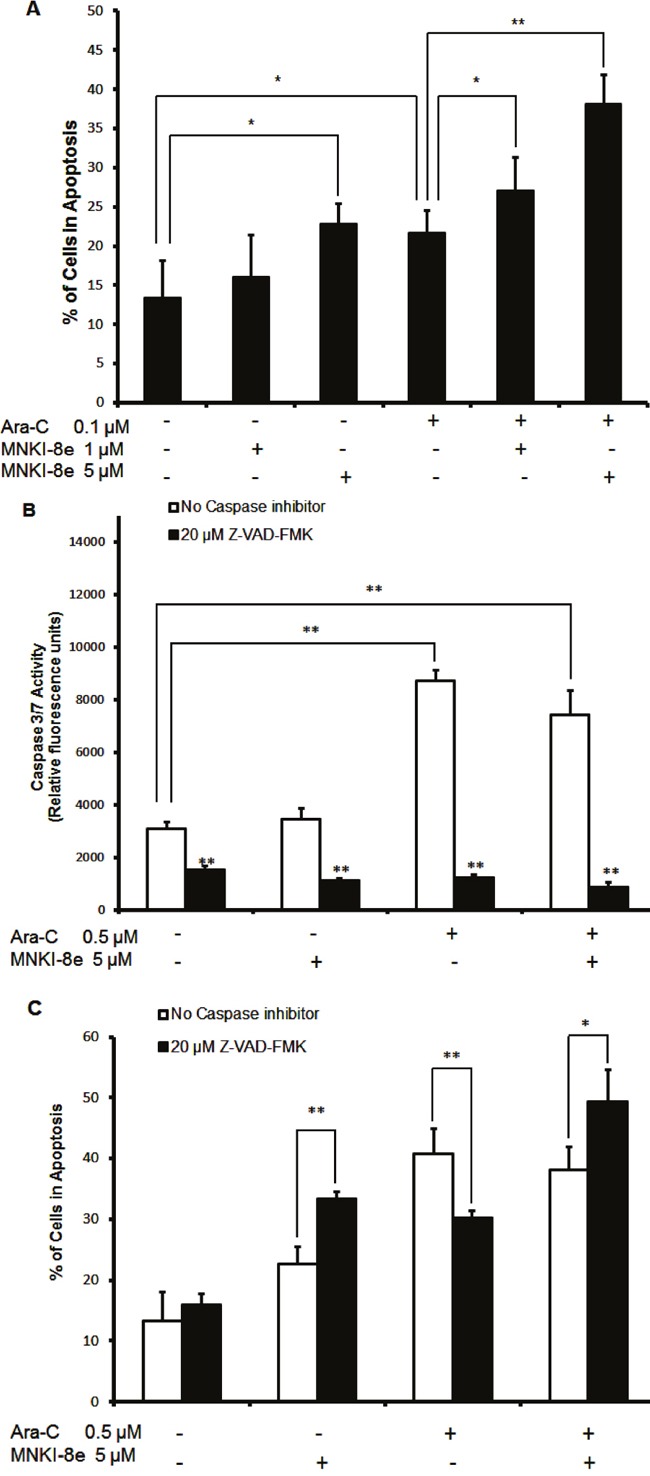
Induction of apoptosis by MNKI-8e, Ara-C or their combination in MV4-11 cells **A.** MV4-11 cells were exposed to 5 μM MNKI-8e, 0.1 μM Ara-C or their combination for 24 h and analyzed by annexin V/PI staining. The percentage of cells undergoing apoptosis was defined as a sum of early (annexin V+/PI-) and late (annexin V+/PI+) apoptotic cells. **B.** Caspases 3/7 activation assay of MV4-11 cells following 24 h treatment with 5 μM MNKI-8e, 0.5 μM Ara-C or their combination in the presence or absence of Z-VAD-FMK (20 μM). **C.** Induction of apoptosis by MNKI-8e and/or Ara-C in the presence or absence of Z-VAD-FMK by annexin V/PI staining. DMSO diluent was used as control in each experiment. Vertical bars represent the mean ± SD of three independent experiments. Data were analyzed by the Student's t-test *p ≤ 0.05 or **p ≤ 0.01.

Induction of apoptosis was assessed with annexin-V FITC/PI double staining of MV4-11 cells after treatment with MNKI-8e or/and Ara-C for 24 h (Figure 2A). Induction of apoptosis was observed with either 5 μM MNKI-8e (23% annexin-V+/PI- plus annexin V+/PI+ cells) or 0.1 μM Ara-C (22% annexin-V+/PI- plus annexin V+/PI+ cells), when compared to untreated MV4-11 cells. The combined treatment of MNKI-8e and Ara-C at molar ratios of 10:1 or 50:1 resulted in increased apoptotic cell death (Figure 2A) compared to the treatment with each drug alone, being consistent with the previous cytotoxicity data shown in Table 2.

We further investigated the activation of caspases 3/7 in MV4-11 cells following exposure to MNKI-8e, Ara-C or their combination for 24 h (Figure 2B). While caspases 3/7 activity was increased at 0.5 μM Ara-C and its combination with MNKI-8e (5 μM; molar ratio = 10:1), no significant effects were observed with MNKI-8e alone. When cells were treated with 20 μM Z-VAD-FMK, a pan-caspase inhibitor, caspases 3/7 activity was reduced to background levels, irrespective of the treatment with Ara-C, MNKI-8e or their combination. In line with these observations, 0.5 μM Ara-C induced apoptosis, which was significantly reduced by 20 μM Z-VAD-FMK (Figure 2C). Surprisingly, exposure to either 5 μM MNKI-8e or its combination with Ara-C (0.5 μM), in the presence of Z-VAD-FMK, increased the apoptotic effects. These results suggested that MNKI-8e enhanced the cytotoxic activity of Ara-C in MV4-11 cells through alternative apoptotic process other than caspases 3/7 activation.

### MNKI-8e inhibits phosphorylation of eIF4E and downregulates Mcl-1 and Bcl-2

**Figure 3:**
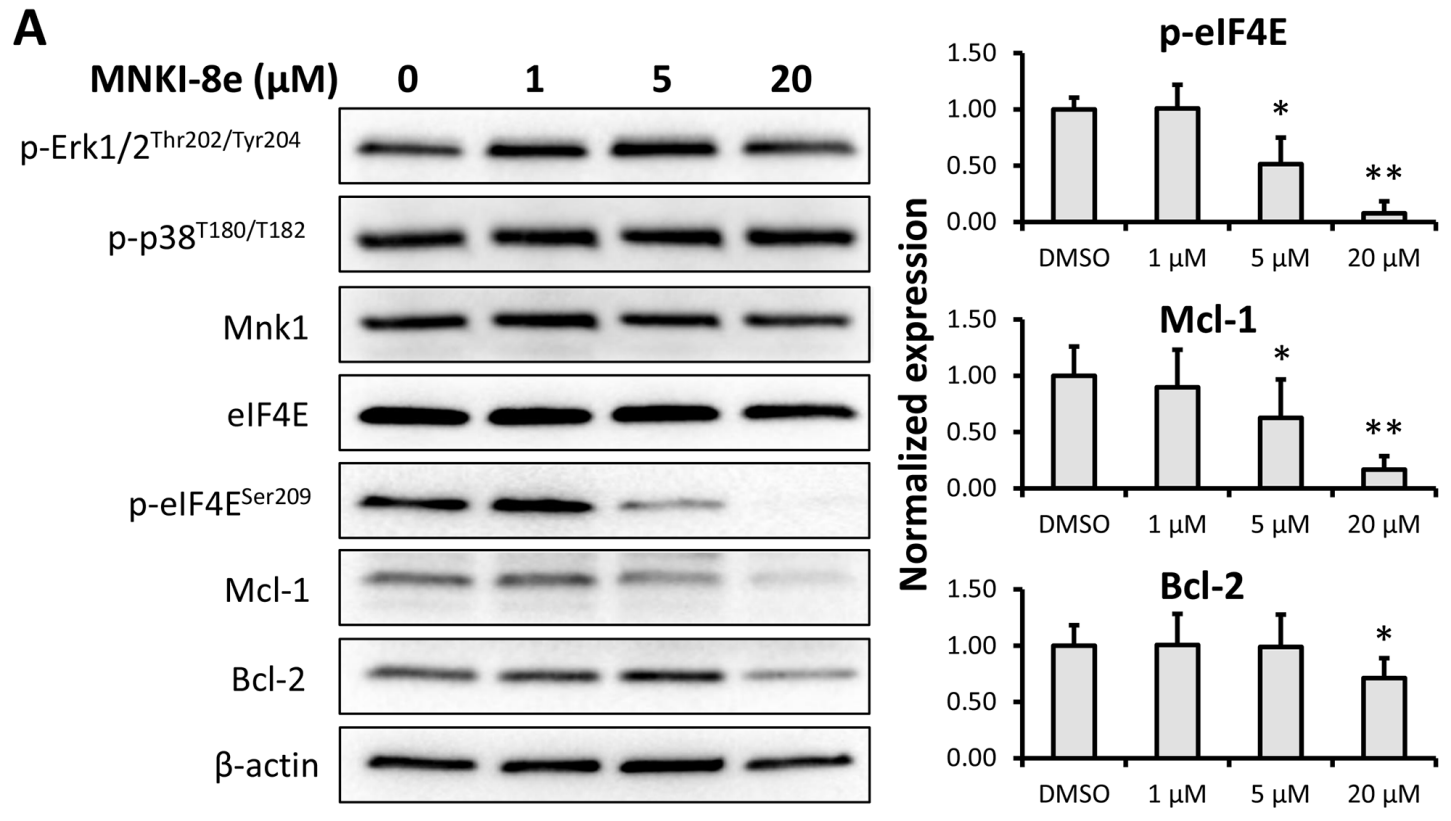

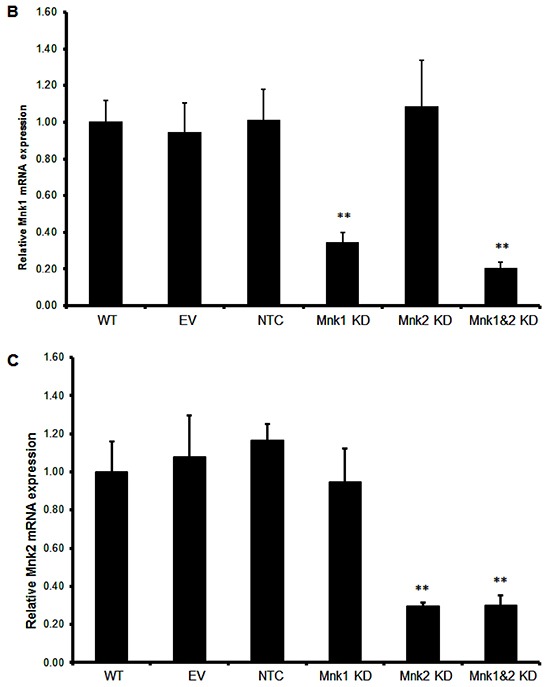

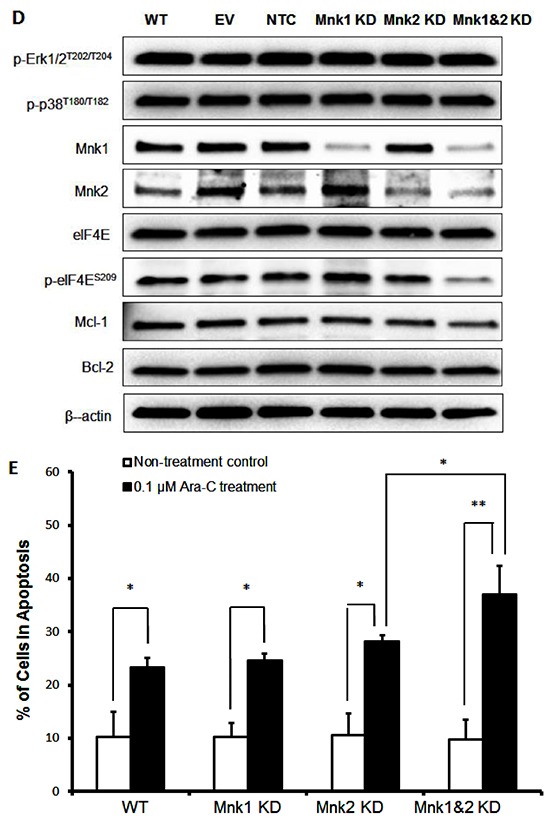
Signaling pathway analysis of Mnk inhibition **A.** MV4-11 cells were exposed to MNKI-8e for 24 h at the concentrations shown and analyzed by Western blotting for the phosphorylation of Erk1/2, p-38 and eIF4E. The expression of anti-apoptotic proteins Mcl-1 and Bcl-2 were also analyzed. β-Actin was used as an internal control. **B.** and **C.** Lentiviral vectors with Mnk1 or Mnk2 shRNA were used to transfect and knockdown mRNA of Mnk1 (*i.e*. Mnk1 KD), Mnk2 (Mnk2 KD) and double knockdown Mnk1&2 (*i.e*. Mnk1&2 KD) in MV4-11 cells. The respective KD levels were confirmed by RT-qPCR when compared to non-transfected MV4-11 cells (WT), the cells treated with empty vector (EV) and non-target control shRNA vector (NTC). Vertical bars represent the mean ± SD of three independent experiments. Data was analyzed by the Student's t-test **p ≤ 0.01. **D.** The effects of Mnk1 KD, Mnk2 KD and Mnk1&2 KD on the MAP kinases and Mnk-eIF4E in MV4-11 cells by Western blot analysis compared to cells treated with EV and NTC. β-Actin was used as an internal control. **E.** Ara-C induction of apoptosis in Mnk1 KD, Mnk2 KD and Mnk1&2 KD cells. The cells were exposed to 0.1 μM Ara-C for 24 h and apoptosis was determined using annexin V/PI assay where annexin V+/PI- plus annexin V/PI+ cells were shown. Data were analyzed by the Student's t-test *p ≤ 0.05 or **p ≤ 0.01. DMSO diluent was used as control in each experiment.

We next investigated the effect of MNKI-8e on MAPK-mediated eIF4E pathways. Western blot analysis of MV4-11 cells following exposure to MNKI-8e for 24 h at different concentrations showed that the level of eIF4E phosphorylation at Ser209 (*i.e*. p-eIF4E^S209^) was reduced at 5 μM and abolished at 20 μM (Figure 3A), confirming cellular Mnk inhibition of MNKI-8e. The levels of phosphorylated Erk1/2 (*i.e*. p-Erk1/2^T202/T204^) and p38 (*i.e*. p-38^T180/T182^) were not affected, suggesting a high selectivity of MNKI-8e for Mnks over their upstream activating kinases. MNKI-8e caused downregulation of the anti-apoptotic proteins Mcl-1 and Bcl-2 in a dose dependent manner in MV4-11 cells.

### Knockdown of Mnk1/2 with shRNA confirms the target specificity of MNKI-8e

To understand the consequences of specific Mnk1 and Mnk2 inhibition, we knocked down Mnk1 and/or Mnk2 expression using a lentiviral short hairpin RNA (shRNA) construct in MV4-11 cells. Stable knockdown of Mnk1 (Mnk1 KD), Mnk2 (Mnk2 KD) or double knockdown of Mnk1 and Mnk2 (Mnk1&2 KD) was achieved to 70-80% compared to empty vector controls analyzed by RT-qPCR (Figures 3B and 3C), and the specificity was also confirmed by Western blot analysis (Figure 3D). As expected, Mnk1 KD, Mnk2 KD and Mnk1&2 KD reduced the levels of respective Mnk1/2 mRNA expression by approximately 70-80%. Phosphorylation of Erk1/2 and p38 were not affected and only Mnk1&2 KD cells showed reduced phosphorylation of eIF4E which was comparable with MNKI-8e treatment at the concentrations ≥ 5 μM (Figure 3A). A modest reduction in Mcl-1 protein level but little change of Bcl-2 expression were observed.

Mnk1&2 KD enhanced the cytotoxicity of Ara-C (*i.e*. >10-fold) in MV4-11 cells compared to Ara-C treatment alone (Table 2), which was in agreement with the observed synergistic effects of MNKI-8e and Ara-C (Figure 1). The induction of apoptosis by Mnk1 KD, Mnk2 KD or Mnk1&2 KD was assessed in combination with Ara-C treatment using the annexin V/PI assay (Figure 3E). Exposure to Ara-C significantly increased the number of apoptotic cells in Mnk1 KD and Mnk2 KD cells compared to wildtype (WT) cells (>10-fold). Mnk1&2 KD cells were more sensitive towards Ara-C and 37% apoptotic cells were detected.

### Ara-C activates MAPK pathways

**Figure 4:**
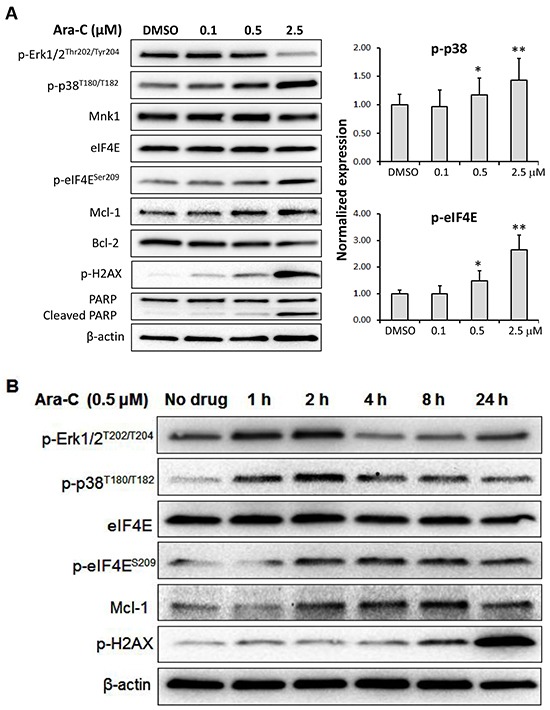
Mechanism of action of Ara-C in MV4-11 cells **A.** MV4-11 cells were treated with Ara-C for 24 h at the concentrations shown. Western blot analysis was carried out with antibodies against phosphorylated Erk1/2, p38, eIF4E and H2AX, as well as Mcl-1, Bcl-2 and PARP. **B.** Western blotting of MV4-11 cells treated with 0.5 μM Ara-C for 1, 2, 4, 8 or 24 h. β-Actin was used as an internal loading control and DMSO diluent was used as control in each experiment.

The effects of Ara-C on phosphorylation of MAP kinases Erk1/2, p38 and eIF4E were further investigated. Treatment of MV4-11 cells with various concentrations of Ara-C for 24 h resulted in enhanced levels of p-p38 and p-eIF4E (Figure 4A). While p-Erk1/2 was reduced with 2.5 μM Ara-C, Mcl-1, Mnk1 and eIF4E were not affected.

It is known that targeting DNA replication is a primary anti-cancer mechanism of Ara-C. Using Western blot analysis, we determined the effect of Ara-C on the phosphorylation of histone 2AX (p-H2AX), a marker of DNA strand breaks, and apoptosis. Ara-C treatment increased the level of p-H2AX in a dose-dependent manner, leading to apoptosis indicated by PARP cleavage (Figure 4A).

To gain a better understanding of the effect of Ara-C on the MAPK pathways, MV4-11 cells were incubated with Ara-C for 1, 2, 4, 8 and 24 h and analyzed by Western blotting as shown in Figure 4B. Interestingly, the levels of p-Erk1/2 and p-p38 were increased after treatment with 0.5 μM Ara-C for 1 h, and reached their highest levels at 2 h post-treatment. The level of p-Erk1/2 dropped to pre-treatment levels after 4 h of exposure, while the level of p-p38 remained elevated in comparison to pre-treatment. The expression of p-eIF4E, Mcl-1 or p-H2AX was increased with Ara-C treatment over the entire time course (Figure 4B).

### MNKI-8e blocks the pro-survival signaling pathways induced by Ara-C

**Figure 5:**
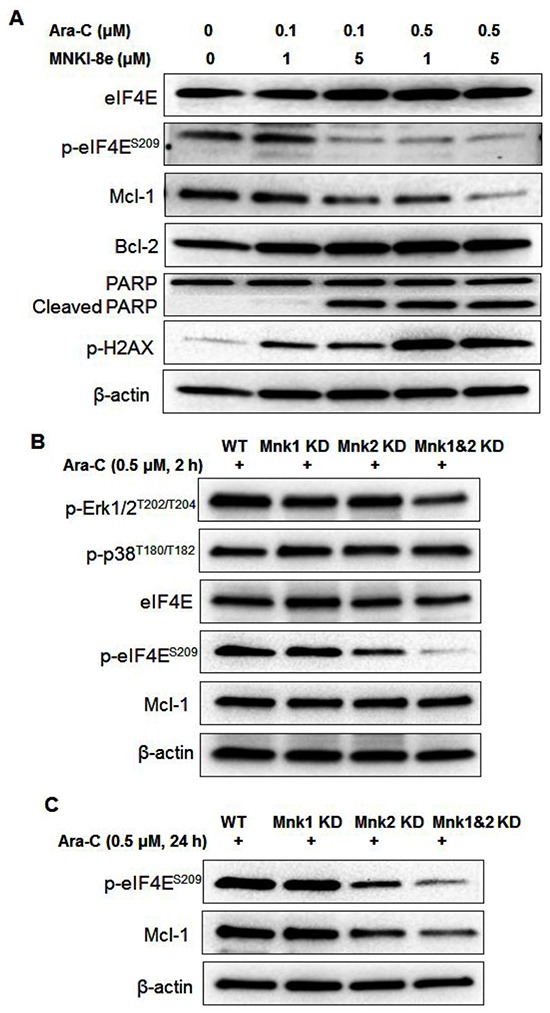
Mnk inhibition enhances the apoptotic activity of Ara-C **A.** MV4-11 cells were treated with MNKI-8e (1 and 5 μM, respectively) and Ara-C (0.1 and 0.5 μM, respectively) for 24 h and levels of proteins including eIF4E, Mcl-1, Bcl-2, PARP and H2AX were assessed by Western blotting. **B.** Western blot analysis of Mnk1 KD, Mnk2 KD and Mnk1&2 KD cells after exposure to 0.5 μM Ara-C for 2 h and **C.** 24 h. β-Actin was used as an internal loading control and DMSO diluent was used as control in each experiment.

We further investigated the underlying mechanisms of synergism between MNKI-8e and Ara-C in MV4-11 cells. As shown in Figure 5A, the combined treatments with MNKI-8e and Ara-C (*i.e*. molar ratio = 10:1) reduced p-eIF4E and Mcl-1 and induced PARP cleavage, but had minimal effects on eIF4E and Bcl-2. The induction of p-H2AX was as expected and consistent with the Ara-C primary mechanism of action.

Mnk1 KD, Mnk2 KD and Mnk1&2 KD cells treated with 0.5 μM Ara-C for 2 h showed minimal effects on p-Erk1/2 and p-p38 proteins. Reduced p-eIF4E level was observed in Ara-C treated Mnk2 KD and Mnk1&2 KD cells, compared to that in Mnk1 KD and WT cells (Figure 5B). Longer exposure to Ara-C, *i.e*. 24 h, led to a reduced Mcl-1 level as shown in Figure 5C, which was consistent with the synergistic effects observed with the co-treatment of Ara-C and MNKI-8e.

## DISCUSSION

Ara-C is an important chemotherapy drug for AML treatment [1, 2]. It is believed that Ara-C enters cancer cells *via* the human equilibrative nucleoside transporter 1 (hENT1), and is subsequently phosphorylated to cytarabine-triphosphate (Ara-CTP) by deoxy-cytidine kinase (dCK) [23, 24]. Ara-CTP incorporates into cell DNA where it reduces DNA elongation and induces breaks in the DNA strand, thereby interfering with DNA replication, leading to apoptosis. However, drug resistance develops in patients with a prolonged treatment of Ara-C leading to treatment failure and high relapse rate [1, 2]. The resistance has been attributed to the downregulated expression of hENT1 and decreased activity of dCK, resulting in a reduced cellular accumulation of Ara-CTP [25, 26]. In this work, we showed that the activated MAPK signaling pathways could be another mechanism of action involved in the Ara-C resistance. We studied the anti-leukemic activity of MNKI-8e and the mechanism underlying its synergy with Ara-C aiming for future development of an effective treatment option against Ara-C-induced resistance in AML.

MNKI-8e is a potent and selective Mnk inhibitor compared to known Mnk inhibitors, *i.e*. cercosporamide and CGP57380 [9, 17] and has demonstrated potent anti-proliferative activity against a panel of leukemic cell lines, particularly MV4-11 cells (Table 1), but showed no toxicity against non-cancerous WI-38 cells. As a single agent, MNKI-8e targeted cellular Mnk-mediated eIF4E phosphorylation at Ser209 and downregulated Mcl-1 expression (Figure 3), resulting in apoptosis of MV4-11 cells. The cellular target selectivity of MNKI-8e was confirmed by knockdown of Mnk1 and Mnk2 with shRNA.

Despite the capability of inducing apoptosis, MNKI-8e had little effects on caspases 3/7 activity in MV4-11 cells (Figure 2), indicating targeting of other apoptotic pathways other than the caspases 3/7 process. Previous studies reported a caspase independent apoptosis in response to MAPK/mTOR targeting in cancer cells [27, 28]. We thus used Z-VAD-FMK to investigate the apoptotic mechanisms of MNKI-8e and Ara-C. As expected, Z-VAD-FMK inhibited caspases 3/7 activity in MV4-11 cells treated with MNKI-8e or/and Ara-C (Figure 2B). Z-VAD-FMK reduced apoptosis in Ara-C treated cells, in contrast, the treatment with MNKI-8e alone or in combination with Ara-C resulted in increased apoptotic or dead cells (*i.e*. annexin V+/PI+) in the presence of Z-VAD-FMK (Figure 2C). The suppression of caspase-dependent apoptosis by Z-VAD-FMK has previously been associated with induction of necrosis [29], which may explain the above apoptotic effects of MNKI-8e in the Z-VAD-FMK-treated cells.

**Figure 6:**
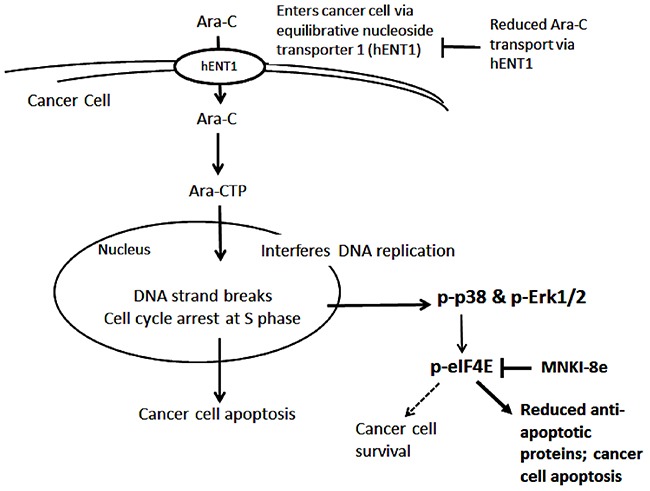
Proposed mechanisms of Ara-C and its combination with Mnk inhibitor After entering cancer cells *via* hENT1, Ara-C is phosphorylated to Ara-CTP, which enters the nucleus, and induces DNA strand breaks leading to apoptosis. On the other hand, Ara-C activates the MAPK-Mnk-eIF4E pathway by inducing the phosphorylation of Erk1/2, p38 and eIF4E, causing an increase in the expression of the anti-apoptotic protein Mcl-1. Resistance to Ara-C treatment is due to increased cancer cell survival which may linked to elevated Mcl-1 expression. Mnk inhibition blocks eIF4E phosphorylation, thereby reducing Mcl-1 protein synthesis and enhancing the anti-leukemic activity of Ara-C.

An earlier report revealed that Ara-C targeted MAPK pathways [8] without clarification of the underlying mechanisms. In this study, we found that Ara-C affected the p38 MAPK leading to the enhanced Mnk kinase activity and thereby Mcl-1 protein (Figure 4). The level of eIF4E was not altered, suggesting that Ara-C had little effect on the mTOR pathways [10, 30]. Our initial data suggested that Erk1/2 phosphorylation was reduced by 24 h treatment with Ara-C at 2.5 μM which was contrary to the previous report [8]. We thus performed a time-course study with Ara-C (Figure 4B). It was revealed that the levels of p-Erk1/2 and p-p38 were rapidly induced and reached their highest levels at 2 h post-treatment. While p-p38 remained higher than its basal level throughout the experiment, p-Erk1/2 was back to pre-treatment levels after 4 h treatment. These suggest that Ara-C triggers a pro-survival mechanism in MV4-11 cells *via* phosphorylation of Erk1/2, p38 and eIF4E, which drives oncogenic translation of Mcl-1 to prevent apoptotic cell death (Figure 6).

This study provides a plausible explanation for the strong synergy between Mnk inhibition and Ara-C. MNKI-8e inhibited Mnk-mediated eIF4E phosphorylation, and thereby blocked Mcl-1 expression leading to enhanced Ara-C induced apoptosis. The mechanism of synergy was further confirmed with Mnk knockdown in MV4-11 cells. The cytotoxicity of Ara-C in the Mnk1&2 KD cells was enhanced by > 10-fold compared to WT cells (Table 2). Likewise, the apoptotic effect of Ara-C was enhanced in Mnk1&2 KD cells (37%) compared to WT cells (23%, Figure 3E). eIF4E phosphorylation and Mcl-1 expression were reduced in Mnk1&2 KD cells after exposure to Ara-C. These results confirm that the MAPK-Mnk-eIF4E pathway plays a critical role in promoting survival of Ara-C-treated MV4-11 cells (Figure 6), and suggests that Mnk inhibition may provide a new therapeutical approach for sensitizing leukemic cells to Ara-C therapy.

## MATERIALS AND METHODS

### Chemicals and reagents

MNKI-8e was synthesized by the Centre for Drug Discovery and Development, University of South Australia, as described previously [17]. Ara-C was purchased from Sigma-Aldrich (Castle Hill, NSW, Australia). Z-VAD-FMK was purchased from Merck Millipore (Bayswater, NSW, Australia).

### Cells and reagents

All cell lines including MV4-11, Kasumi-1, PL-21, KG-1, U937 and WI-38 were obtained from the cell bank at Centre for Drug Discovery and Development (University of South Australia). The cell lines were cultured in RPMI-1640 medium supplied with 10% fetal bovine serum (FBS) (Sigma-Aldrich, Castle Hill, NSW, Australia) within a humidified 37°C, 5% CO_2_ incubator.

### Knockdown of Mnks with lentiviral shRNA in MV4-11 cells

Knockdown of Mnk1 or/and Mnk2 was performed with lentivirus-mediated shRNA particles and verification of the percentage of knockdown was performed as described previously [31]. Lentiviral vector pLKO.1-puro with Mnk1 or Mnk2 shRNA was obtained from Sigma-Aldrich (Castle Hill, NSW, Australia), and an empty vector and a non-target control shRNA vector (Sigma-Aldrich, Castle Hill, NSW, Australia) were used as controls. MV4-11 cells were incubated with lentiviral vectors for 24 h at 37°C. The lentivirus-transduced cells were then selected by culturing with 1 μg/mL of puromycin for two weeks. The knockdown of Mnk1 or/and Mnk2 mRNA was confirmed by RT-qPCR experiments and the reduced expression of Mnk1 or/and Mnk2 proteins was assessed by Western blotting.

### Cell viability assays

Cell viability of leukemia cell lines was performed with the resazurin assay (Sigma-Aldrich, Castle Hill, NSW, Australia) as described previously [17]. Concentrations of compounds required to inhibit 50% of cell viability (GI_50_) were calculated using non-linear regression analysis. The cell viability of WI-38 cells was tested using the MTT assay (Sigma-Aldrich, Castle Hill, NSW, Australia) as described previously [32].

### Evaluation of apoptosis

Apoptosis of cancer cells was detected with an annexin V fluorescein isothiocyanate /propidium iodide (Annexin V/PI) flow cytometry assay as described previously [33]. Briefly, MV4-11 cells were seeded and incubated overnight at 37°C under 5% CO_2_. After treatment with the compounds, the cell pellets were collected and stained using an annexin V/PI commercial kit following the supplier's protocol (Becton Dickinson, Australia). The samples were analyzed with a Gallios flow cytometer (Beckman Coulter, Australia) within 1 h after staining. The data were analyzed using the Kaluza v1.2 software (Beckman Coulter, Australia).

### Caspases 3/7 activation assay

MV4-11 cells were plated in a 96 well plate overnight. Subsequently the cells were exposed to the compounds for 24 h at different concentrations. The caspase activity was determined using an Apo-ONE Homogeneous Caspase-3/7 kit (G7790 Promega, Madison, WI, USA) according to the manufacturer's instructions and analyzed using an EnVision multi-label plate reader (PerkinElmer, Beaconsfield, UK). The experiments were repeated at least three times to assess statistical relevance.

### Western blots

Western blotting was performed as described previously [34, 35]. Antibodies used were as follows: p-Erk1/2, p-p38, Mnk1, eIF4E, p-eIF4E, Bcl-2, Mcl-1, PARP, cleaved PARP, p-H2AX, β-actin (all aforementioned antibodies from Cell Signalling Technology, Danvers, MA, USA), Mnk2 (Abcam, MA, USA), and Bcl-2 (Dako, Glostrap, Denmark). Both anti-mouse and anti-rabbit immunoglobulin G (IgG) horseradish peroxidase-conjugated antibodies (Dako, Glostrap, Denmark) were used as secondary antibodies. Enhanced Chemiluminescence (ECL) reagents (GE Life Sciences, Australia) were used for detection.

### Real time-quantitative PCR (RT-qPCR)

RNA was extracted from the cells using a High Pure RNA Isolation Kit (Roche Applied Science, Castle Hill, NSW, Australia) and 1 μg of RNA was used in a 20 μL reverse transcription reaction (Transcriptor First Strand cDNA Synthesis Kit, Roche Applied Science, Castle Hill, NSW, Australia). RT-qPCR was performed in duplicate using SYBR Green I dye (Roche Applied Science, Castle Hill, NSW, Australia) with the LightCycler LC96 (Roche Applied Science, Penzberg, Germany). All primers were purchased from Sigma-Aldrich (Castle Hill, NSW, Australia). Relative quantification using E-method established by Roche Applied Science was performed with β-actin mRNA as reference sequence and untreated samples as study calibrator. The cDNA samples were amplified using the following primer pairs with amplification efficiency (E): β-actin: 5′-ACTCTTCCAGCCTTCCTTC-3′(forward) and 5′-GATGTCCACGTCACACTTC-3′(reverse), E = 1.70; Mnk1: 5′-AAGGCCATTGAGACACTTCG-3′(forward) and 5′-CCCAAATGAAATAAAGCTCCTG-3′(reverse), E = 1.74; Mnk2: 5′-TCCTGCAGAGGTGGGACAGT-3′(forward) and 5′-ACGGTTCTGACCAGTCCTCC-3′ (reverse), E = 1.75.

### Statistical analysis

All experiments were performed with at least two independent repeats and variations about mean were presented as standard deviations. Statistical comparisons were performed using Student's *t*-test and *p* ≤ 0.05 were considered significant.
